# Cause of the Failure of Vaccination in France

**Published:** 1846-11

**Authors:** 


*Cause of the failure of Vaccination in France*—M. Testel adopts the opinion, that the vaccine virus is liable to undergo degeneration, and therefore requires to be occasionally renewed. He thinks, however, that the facts admit of an explanation entirely different from that which is generally received. He endeavours to show that the distinction hitherto made between genuine and spurious vaccination is insufficient, and that an intermediate state exists. He calls this a mixed form of vaccination, in which there is a *preservative power* for a longer or a shorter period, and in a greater or less degree. In his view the vesicle formed under these circumstances, has been hitherto confounded with the true vaccine vesicle, and from this has arisen the opinion that the vaccine virus has undergone degeneration and that revaccination is necessary.—*Lond. Med. Gaz., from Comptes Rendus.*

